# A plant-like battery: a biodegradable power source ecodesigned for precision agriculture†

**DOI:** 10.1039/d2ee00597b

**Published:** 2022-05-30

**Authors:** Marina Navarro-Segarra, Carles Tortosa, Carlos Ruiz-Díez, Denis Desmaële, Teresa Gea, Raquel Barrena, Neus Sabaté, Juan Pablo Esquivel

**Affiliations:** Instituto de Microelectrónica de Barcelona, IMB-CNM (CSIC) C/dels Tillers sn, Campus UAB 08193 Bellaterra Barcelona Spain juanpablo.esquivel@csic.es; Universitat Autònoma de Barcelona (UAB) 08193 Bellaterra Barcelona Spain; Catalan Institution for Research and Advanced Studies (ICREA) Passeig Lluís Companys 23 08010 Barcelona Spain; BCMaterials, Basque Centre for Materials, Applications and Nanostructures, UPV/EHU Science Park 48940 Leioa Spain juanpablo.esquivel@bcmaterials.net; IKERBASQUE, Basque Foundation for Science 48009 Bilbao Spain

## Abstract

The natural environment has always been a source of inspiration for the research community. Nature has evolved over thousands of years to create the most complex living systems, with the ability to leverage inner and outside energetic interactions in the most efficient way. This work presents a flow battery profoundly inspired by nature, which mimics the fluid transport in plants to generate electric power. The battery was ecodesigned to meet a life cycle for precision agriculture (PA) applications; from raw material selection to disposability considerations, the battery is conceived to minimize its environmental impact while meeting PA power requirements. The paper-based fluidic system relies on evaporation as the main pumping force to pull the reactants through a pair of porous carbon electrodes where the electrochemical reaction takes place. This naturally occurring transpiration effect enables to significantly expand the operational lifespan of the battery, overcoming the time-limitation of current capillary-based power sources. Most relevant parameters affecting the battery performance, such as evaporation flow and redox species degradation, are thoroughly studied to carry out device optimization. Flow rates and power outputs comparable to those of capillary-based power sources are achieved. The prototype practicality has been demonstrated by powering a wireless plant-caring device. Standardized biodegradability and phytotoxicity assessments show that the battery is harmless to the environment at the end of its operational lifetime. Placing sustainability as the main driver leads to the generation of a disruptive battery concept that aims to address societal needs within the planetary environmental boundaries.

## Abstract 2

A biodegradable battery inspired by the transpiration pull of liquids in plants has been ecodesigned to power wireless sensors and then be safely biodegraded or composted, resembling the way a plant comes back to nature at the end of its lifecycle.

## Graphical abstract

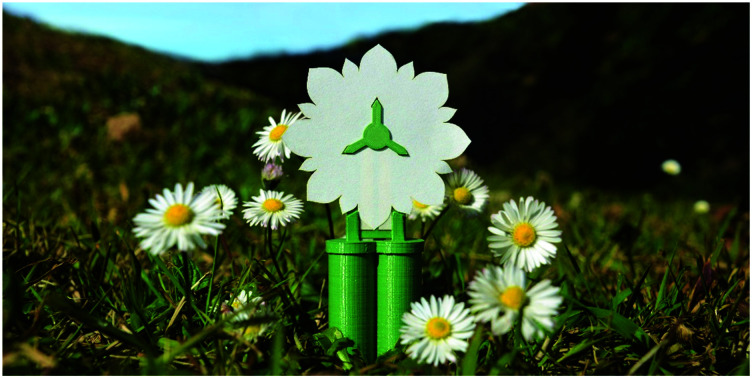

**Broader context**

Waste electrical and electronic equipment (WEEE) or e-waste is becoming the fastest growing waste stream worldwide. Due to their content of heavy metals and strong electrolytes, batteries are one of the most hazardous components of e-waste. Regrettably, the vast majority of the batteries sold worldwide are neither properly disposed nor recycled after use. The demand for portable batteries is expected to increase significantly in the following years due to the upcoming wave of power hungry Internet-of-Things (IoT) devices. Agriculture is a strategic sector that particularly demands electronic devices to increase the efficiency of food production while minimizing the use of agrochemicals. This demand for portable energy must be fulfilled by power sources with high performance and also they should possess environmental sustainability in terms of their materials, fabrication methods and disposal. This work presents a nature-inspired battery, specifically ecodesigned to follow the lifecycle for applications in precision agriculture. The proposed battery meets the power requirements for wireless sensors and is composed of benign non-toxic materials that undergo biodegradation at the end of its operational life cycle.

## 1. Introduction

Agriculture needs a new paradigm. With the worldwide population predicted to reach 9 billion inhabitants by 2050,^1^ it is likely that the overall food demand will continue to grow. To meet this demand, ever-increasing crop yields are required. Nevertheless, it is today widely recognized that the intensive use of agrochemicals (*e.g.*, fertilizers, herbicides, pesticides, *etc.*) that have been utilised so far to boost food production is not sustainable in the long term. Intensive agriculture based on high inputs of chemicals proves to be one of the major drivers of soil degradation, pollution of natural water resources, and biodiversity loss. In this regard, wireless sensor networks (WSNs) can readily help growers to adapt their practices and shift towards precision agriculture (PA).

In a nutshell, WSNs for PA are based on a collection of individual nodes deployed across agricultural lands to monitor a variety of local parameters (*e.g.*, air temperature, soil moisture, soil PH, salinity, light, *etc.*). Measurements are then used to derive microclimate conditions and/or detect variability among different parcels. This way, instead of treating uniformly (and sometimes unnecessarily) a whole field, growers can better apply the right amount of inputs, at the right place and at the right time. To the best of our knowledge, however, WSNs for PA are deployed in a limited number of agricultural settings; mainly those whose configuration remains relatively unchanged from season to season (*e.g.*, vineyards,^2,3^ olive groves^4^ or orchards^5,6^). Comparatively, there is a scarcity of studies reporting the use of WSNs in cereal crops (*e.g.*, wheat, millet, quinoa, *etc.*). From our personal analysis, a plausible main reason that hampers a wider adoption of WSNs in cereal crops is that the nodes may actually constitute obstacles to be avoided during harvesting. Recollecting tens or even hundreds of nodes before harvesting, however, is not a plausible option. In the future, low-cost nodes that could just be left in the soil indefinitely without negatively impacting the environment could bring innovative solutions to such limitations. Nevertheless, there are several technological challenges to be tackled to turn such an ideal case scenario into reality. One of the major hindrances is that current nodes are far from being eco-friendly. In particular, the power source turns out to be a major source of potential pollution. At present, most nodes in PA are battery-powered. Commercially available batteries, however, are loaded with heavy metals and/or toxic chemicals (*e.g.*, lithium, cadmium, manganese, copper, zinc, *etc.*) which prove to be highly detrimental to the environment.^7^ The use of solar panels, wind generators, or hydro-generators connected to irrigation pipes was reported for supplying power to nodes in PA.^8^ Nonetheless, such energy harvesting devices cannot be used on their own. Indeed, they solely produce electrical energy sporadically (*i.e.*, if it is windy, if it is not too cloudy, *etc.*). As a direct consequence, they actually serve exclusively as auxiliary recharging units to prolong the batteries’ lifespan. Since conventional batteries are still needed, the situation is actually even worsened from the point-of-view of the environmental impact. For instance, solar panels also include a variety of hazardous materials.^9,10^ Generally speaking, the end-of-life of electrical electronic equipment (*i.e.*, their disposal, dismantling, difficult and limited recycling) has already started to arise alarming societal concerns. The deployment of new digital technologies in PA should not come at the price of an additional uncontrollable electronic waste burden. Because batteries are considered as one of the major sources of e-waste, novel sustainable power sources are needed to enable a more massive deployment of WSNs in PA.^11^

In the context of PA, new battery architectures featuring nature-based characteristics appear especially appealing. This is the case for microfluidic galvanic cells (μGCs). We here refer to μGCs as miniature fluidic devices that can produce an electric current by means of a redox reaction, such as membraneless microfluidic fuel cells (μFCs) or microfluidic redox flow cells. One of the great advantages of μGCs is that they can generate electrical energy using a variety of reactants, including biofuels.^12–16^ For instance, μFCs exploiting methanol, ethanol, or glucose as fuel have been reported.^17–25^ The possibility of driving low-consumption electronic systems, including wireless sensors, was also demonstrated.^26^ μFCs have thereby been envisioned as promising candidates to replace small conventional primary batteries. Unfortunately, even after almost two decades of intense research efforts, μFCs have not succeeded in leaving the lab bench yet. The problem is that syringe pumps or pressure controllers are required to drive the reactants towards the electrochemical reactive zones inside the μFCs. Such auxiliary external pieces of equipment are bulky and expensive. Even worse is that they may consume more electrical energy than the MFCs can actually produce. To eliminate external pumping systems, the scientific community has come up with μFCs made of paper materials. Paper can indeed serve as a low-cost passive pumping mechanism because it can absorb and drive liquids through capillary action.^27,28^ Paper-based materials are even more appealing for PA because they are nature friendly (*e.g.*, cellulose can be made of fibers coming from corn straw, bamboo, bagasse, grass-plants like reeds, *etc.*). Nonetheless, although paper offers a very simple and elegant solution to bypass the use of external pumps, many paper-based μFCs still make use of critical raw materials to produce electricity (*e.g.*, platinum, palladium, platinum-ruthenium alloys, *etc.*).^15,29^ The use of such noble metal catalysts does not align well with an ideal circular sustainable approach. Although a paper-based μFC using enzymes as biocatalysts was reported to be capable of driving a digital clock,^30^ the well-recognized restricted stability of enzymes over time appears as a strong limiting factor for targeting real PA applications. Moreover, enzymes are often most active solely in a precise range of temperature.^31,32^ However, unlike in indoor environments, strong variations under ambient conditions over days/nights (*e.g.*, temperature, humidity, *etc.*) must be expected in an open field. In 2017, Esquivel *et al*. reported PowerPAD3.^33^ Different from the paper-μFCs previously reported, the completely all-organic PowerPAD architecture did not involve the use of any metals or enzymes. Notably, the authors assessed and validated that the battery could be biotically degraded by microorganisms. This represented a significant step forward, since it means that the battery could be discarded in a field with a minimal environmental impact. Since then, several other examples of batteries focusing on biodegradation as the alternative end-of-life have been reported.^34–36^ A limitation of the PowerPAD, however, was that electricity was produced mainly through a diffusion process which limited the overall battery efficiency. More recently, the same group reported an enhanced design where the battery performance was increased by exploiting quasi-steady capillary flows. Despite the gain obtained in performance, the operational lifetime remained limited; in both designs, the batteries could generate electric energy only for a few hours. Unfortunately, this is too restrictive for targeting PA applications.

**Fig. 1:**
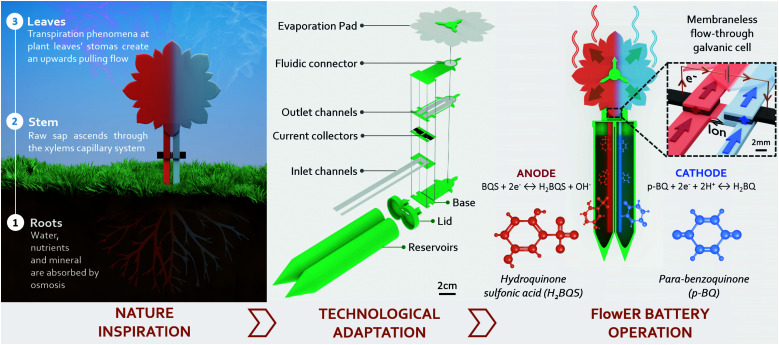
FlowER battery concept evolution from the inspiration, found in the liquid transport in plants, to an operational prototype. The technological adaptation of the FlowER battery concept has been performed through the selection of environmentally harmless materials to produce two inlet channels acting as roots, from which the device nurtures with dissolved redox species, just like plants take water and nutrients from their surroundings. The main laminated paper core, analogous to a plant stem, includes two porous carbon current collectors and a salt bridge, indispensable components to create a membraneless galvanic cell. The core ends with two outlet channels that transport the fluid towards the top. A rounded leaf-like absorbent pad exposed to the atmosphere keeps wicking the solution by transpiration pull through the battery. Finally, a 3D printed compostable green casing (*i.e.*, reservoirs + lid + base + paper supports) was used to provide mechanical robustness and verticality.

Herein, we introduce the FlowER: an Evaporation Flow Redox battery intended to bring new solutions to some of the challenging requirements imposed by PA applications (Fig. 1). The FlowER battery conception and development approach invokes a change in the current portable battery models. This change pursues a new battery paradigm in which power sources are designed to be paired with the life cycle of the intended application. Environmental sustainability has been placed as the core priority to create a disruptive technology that can evolve within the environmental boundaries of the planet. In this process, eco-design was used as a fundamental tool, from raw material selection to disposability considerations – including manufacturing processes, operational time, and energy capacity – to create a prototype that meets PA-lifecycle requirements, namely, affordability, extended operational lifespan, and biodegradability. The overall architecture of this new battery is profoundly inspired by nature: both its appearance and the way it operates make it a true “flower-like” battery. The “roots, stem and petals/leaves” are all made of low-cost paper-based materials. An absorbent pad, placed on the top of the FlowER, exploits evaporation combined with cohesion and capillary forces in a way analogous to transpiration pull in plants. Liquid reactants contained in reservoirs can thereby flow continuously through porous carbon electrodes where electrochemical reactions take place. Notably, the operation time, which usually ranges from solely tens of minutes to a few hours maximum in conventional paper-based fuel cells, can be significantly extended. Furthermore, the harmless green chemicals selected prove to be powerful enough for real-world applications. As a proof-of-concept, we demonstrate that the FlowER can supply power to a commercial wireless monitoring unit dedicated to precision horticulture. Finally, the possibility for the new plant-inspired battery to end its life cycle by returning to nature is assessed by standardized ecotoxicity and biodegradation tests.

## 2. Results and discussion

### 2.1 Fundamentals of water transport in plants

All living organisms need to transport fluids to survive and prosper. In the animal kingdom, many species rely on a heart to ensure blood circulation within the body. In contrast, plants are not equipped with analogue contractile organs. Yet some species of trees are able to transport fluids upwards over incredibly long distances (*e.g.*, some sequoia trees can be more than 100 m high^37^). Strikingly, they do it against gravity and in a passive way, namely, without dedicating metabolic energy to pumping. Instead, they rely on a combination of three intertwined passive flow mechanisms.

The plant roots first capture the water found in the surrounding soil. Then, an osmotic-flow mechanism generates a positive pressure that pushes the water collected towards the xylem. The xylem is a portion of the sapwood composed of repeating narrow vessels that run vertically up the plant. This vascular tissue is made of the hollow walls of dead cell skeletons, and is mainly involved in the ascent of bulk – almost pure – water from the roots up to the leaves.‡‡For a more exhaustive description about plant physiology (*e.g.*, consideration of the phloem tissue, *etc*.) the interested reader may consult dedicated review articles (*e.g.*, see 81 and references therein). Once the water has reached the xylem conduits, capillarity comes into play. Indeed, depending upon the species and location of the plant, the inner diameter of the xylem conduits can range from 10 μm to *ca.* 100 μm. Nevertheless, capillary rise varies with the inverse of the vessel diameter. As discussed by Steudle, even with the smallest xylem conduits, water can rise not more than 3 m high when considering solely capillary effects.^38^ It is the capillarity-transpiration combination that is actually considered to be the fundamental mechanism of water transport in large multi-cellular plants. Transpiration (*i.e.*, the conversion of water from liquid to gas) is predominant in the foliage where water molecules evaporate through the multitude of closable microscopic slit-like pores (stomata) mainly present on the undersides of leaves. The sun provides the energy to overcome the latent heat of evaporation of the water molecules. The removal of the water molecules from the water–air interface (*i.e.*, menisci) cause the interfaces to recede into the pores and change their shape^39^ (*i.e.*, the menisci become more concave). Capillarity acts to restore the initial equilibrium shape of the menisci, generating a strong pulling force so that water lost during the evaporation process is replaced.

Interestingly, several engineering approaches have tried to capture the main attributes of the xylem and/or the transpiration mechanism occurring in plants to create miniature synthetic devices capable of pumping fluids without the use of any moving mechanical parts.^40–44^ Importantly, one could envision to use such miniature pumps in conjunction with μFC architectures in order to partly solve some of the aforementioned problems (*e.g.*, bypassing the use of conventional bulky syringe pumps). Nonetheless, advanced microfabrication techniques and/or non-environmentally friendly materials were still mostly needed to fabricate these devices. As an alternative, porous materials with pores in the micrometer-size range have been shown to be appropriate for efficient capillary pumping of water. This is the case for paper-based materials. Furthermore, in a manner similar to plants, paper-based materials can take advantage of both capillary and evaporation effects.^28,45^ Because the large deployment of numerous nodes for high resolution measurements capabilities in PA will require low-cost devices employing environmentally responsible materials, we selected paper as the building block for the fabrication of our FlowER battery.

### 2.2 Device design and operation principle

A evaporation-driven flow battery has been designed with a plant-inspired operation principle, indicating that it has two inlet channels acting as roots, from which the device nurtures with already dissolved redox species, just like plants take water and nutrients from their surroundings. The main laminated paper core, analogous to a plant stem, includes two porous carbon electrodes connected by a U-shape paper that acts as a salt bridge, indispensable components to create a membraneless microfluidic galvanic cell. The core ends with two outlet channels that transport the fluid towards the top of the device. Finally, a rounded leaf-like absorbent pad exposed to atmosphere keeps wicking the solution by transpiration pull through the core (see Fig. 1).

The prototype has been designed to be able to be planted in the bare ground. In a nutshell, a 110 mm × 12 mm U-shaped piece of paper creates the inlet channels for the solutions and also forms the salt-bridge. Two porous carbon electrodes of 5 mm × 10 mm create a flow-through electrode configuration and connect to two separate outlet-channels of 40.5 mm × 12 mm (defining a geometrical electrode area of 0.25 cm^2^). The fluidic system ends up in the center of a flower-shaped absorbent pad that absorbs liquids omnidirectionally around its 360°, maximizing the evaporation exposed area while keeping a co-laminar flow. Fan-shaped absorbent pads were first proposed by Mendez *et al.* in 2010, proving that the gradual increase of the unwetted area induces a quasi-steady flow rate in the preceding channel sections.^46^ More recently, our group incorporated this fluid control mechanism in a paper-based redox flow battery.^47^ The quasi-steady flow translated into a stable power delivery over time. When working under evaporation conditions the absorbent pad will be called evaporation pad. The paper assembly is fed with redox species solutions already dissolved in a 15 mL volumetric capacity reservoir. In order to create the final prototype, the battery paper structure is coupled with a 3D printed compostable casing, which provides verticality and mechanical robustness and compensates the thickness of the different paper layer (a photograph of all the device components is shown in Fig. S1, ESI†).

Using the evaporation action to drive the battery's main flow is, for paper microfluidics, the next natural step towards autonomy and competitiveness. Making use of a transpiration mechanism instead of capillary forces allows the system to become independent of the absorbent volumetric capacity. Hence, the operation time is also decoupled from the paper's total volumetric capacity (137 μl for this particular shape system), but instead depends on the volume of the reactant reservoirs (15 mL in this case). On the other hand, the fluid flow rate changes its dependence from the absorbent pad material wicking properties to the evaporation-exposed area, becoming constant (under certain atmospheric conditions) and easily tunable. Combining these two characteristics, the result is a miniaturized low-cost and paper-based autonomous mid-term operational battery (from hours to days), which can start to compete in operational time terms with traditionally commercial ones, thus bringing paper-based microfluidics power suppliers up to the next level.

### 2.3 Influence of the evaporation pad area on the evaporation flow rate

Flow rate (*Q*) is one of the key parameters that determine a flow battery performance, as it defines the reactants availability for electrochemical conversion. In the particular case of the device herein presented, evaporation-driving forces govern the flow rate. Even though, the paper structure fills up by capillary forces, once completely full, the FlowER battery takes advantage of the transpiration mechanism naturally occurring on the evaporation pad area to pull fresh redox species through the electrodes and clean the reaction products, keeping an active flow towards the top.

**Fig. 2:**
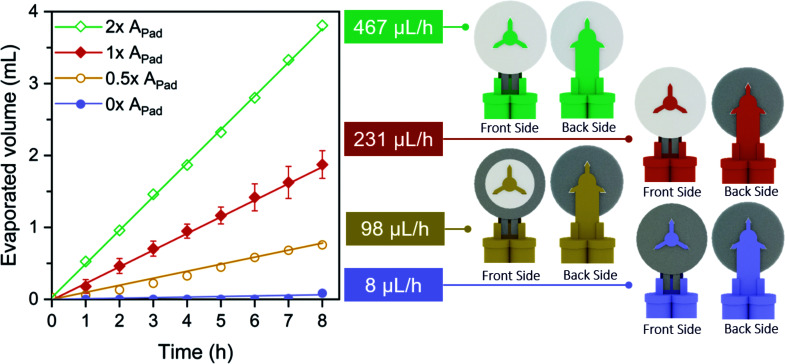
Evaporation flow rate control related to the evaporation area exposed to the air: 2×, 1×, 0.5× and 0× times the area of one side of the evaporation pad (*A*_Pad_ = 19.6 cm^2^). Linear fit adjustment coefficients: *R*_2×*A*_^2^ = 0.9997, *R*_1×*A*_^2^ = 0.9996, *R*_0.5×*A*_^2^ = 0.9924 and *R*_0×A_^2^ = 0.9427. Environmental conditions: HR% ∈ [51, 56]%, *T* ∈ [28, 30] °C and *P* = 1 atm.

The evaporation phenomenon has been widely studied in paper microfluidics due to the direct influence it may have on precision and reproducibility of the devices.^48–51^ Here, a study was carried out to investigate how to control and adapt transpiration pull to the battery requirements. The evaporation pad area (A_pad_) is an easily tunable parameter that determines the total liquid–air interface where the evaporation phenomenon takes place, and hence the evaporation flow rate (*Q*_evap_) generated through the fluidic system. Four different *A*_Pad_ configurations were tested (2 × *A*_Pad_, 1 × *A*_Pad_, 0.5 × *A*_Pad_, and 0 × *A*_Pad_), by covering different percentages of the evaporation pad area, 1 × *A*_Pad_ being the area of one side of the absorbent pad (19.6 cm^2^). A blue dye was used to visualize the total volume of liquid evaporated from the reservoirs through the fluidic system over time. As shown in Fig. 2, the evaporation phenomenon at the absorbent pad creates a quasi-steady flow (*i.e.* a constant slope over time) for 8 consecutive hours. Furthermore, the volume of the liquid evaporated at a given time increases proportionally while increasing the *A*_Pad_. Thus, under the same environmental conditions, the ratios between the evaporation flow rates (467 μL h^−1^, 231 μL h^−1^, 98 μL h^−1^, and 8 μL h^−1^) and the corresponding evaporation exposed areas 2 × *A*_Pad_, 1 × *A*_Pad_, 0.5 × *A*_Pad_, and 0 × *A*_Pad_ are equal.

The flow rates, reported previously by our group, obtained with a capillary-driven fluidic system, were 900 μL h^−1^, 540 μL h^−1^ and 288 μL h^−1^ depending on the outlet channel length,^47^ with operational times of 7, 13 and 25 minutes, respectively. Comparing both fluidic systems, it can be concluded that using evaporation as a driving force, the flow rates generated are within the same order of magnitude than those generated by capillarity. Finally, it has to be noticed that, by completely covering the absorbed pad area, 0 × *A*_Pad_, a negligible evaporation flow was obtained, indicating that no other evaporation sites are created than the ones desired on the evaporation pad.

### 2.4 Influence of the environmental conditions on the evaporation flow modeling

As mentioned above, the power delivered by the battery depends on the flow rate. Hence, for the purpose of this work it was necessary to fully understand the influence of environmental conditions on the evaporation flow rate. For this reason, a simple evaporation flow rate modeling was proposed (the complete mathematical development is presented in the ESI†). Eqn (1) shows the dependencies and parameters that influence the generated evaporation flow [g s^−1^],1
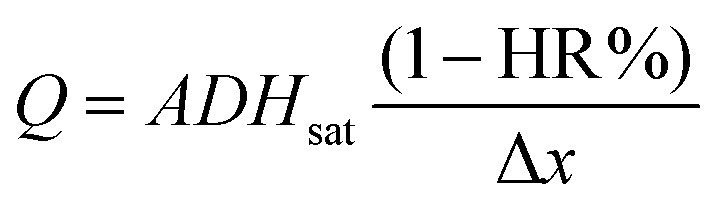
where *D* [m^2^ s^−1^] is the diffusion coefficient of the liquid, Δ*x* [m] represents the distance between 2 points in space, *A* [m^2^] is the exposed area (*i.e.* total surface of liquid–air interface), *H*_sat_ [g m^−3^] is the saturation humidity (*i.e.*, total amount of water that can be dissolved in the dry air at a given temperature), and HR% is the relative humidity (*i.e.*, amount of water dissolved in the air in percentage respect to the saturation humidity). Here, *D* is approximated to 25.6 × 10^6^ m^2^ s^−1^ (*T* = 20 °C) and Δ*x* is set as 10^−6^ m to normalize the orders of magnitude of experimental and theoretical flow rates.

**Fig. 3:**
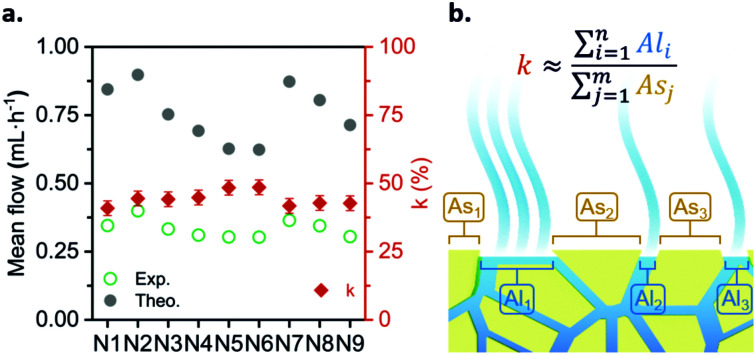
Theoretical *vs.* experimental evaporation flow rates and the corresponding ratio, *k* (%), between each of them. *N* = 9 repeats.

In order to validate the proposed *Q*.evap modeling, the experimental evaporation flow rates presented in the previous section were compared with the theoretical flow rates estimated using eqn (1). The validation was done with the 2 × *A*_Pad_ configuration, where *A* = 35.5 cm^2^. Ambient temperature and relative humidity values were recorded with a sensor during the set-up course. Fig. 3a shows the comparison between the experimental and theoretical flow rates for nine repeats. As it can be observed, both values significantly differ; however, when computing the percentage difference between theoretical and experimental values, a constant *k* = 44.3 ± 2.7% is obtained.

The disagreement between the experimental and theoretical flows, expressed in the *k* parameter, could be explained due to a discrepancy in the exposed evaporation area. The parameter *A* in eqn (1) stands for the total liquid–gas interface; taking into account that in this case a paper-based evaporation pad is being used, the geometrical area does not correspond with the evaporation exposed area (see Fig. 3b). Due to the physical space occupied by the paper fibers, the actual liquid–gas interface area is lower than the geometric one. The *k* ratio compensates for this disagreement and other collateral effects of using a specific porous material as an evaporation pad (*i.e.* adhesion forces generated between the liquid and the paper walls). This ratio also allows the calculation of the actual flow rate of the paper-based fluidic system directly from pre-recorded environmental conditions (*T*°, HR%, *P*). The theoretical modeling of the evaporation flow rate eliminates the need for photographic analysis, deepens the environmental conditions’ influence comprehension, and makes it possible to predict the device performance in different climates and environments. Henceforth, all the prototype characterization experiments were conducted while simultaneously measuring ambient parameters with a commercial sensor. The recorded environmental data provide information about the battery working conditions and allow the computation of the actual working *Q*_evap_, strengthening the prototype control during characterization. Furthermore, it allows the prediction of the battery behavior in terms of *Q*_evap_, for a wide range of ambient conditions. Fig. S3 (ESI†) depicts the hygrometric chart relating the battery theoretical flow rate with temperatures from 5 °C to 35 °C and relative humidity conditions from 0% to 90%.

### 2.5 Battery operation and optimization

Once the paper structure was fluidically validated, the configuration creating the highest flow rate (*Q*_evap_ = 467 μL for 2 × *A*_Pad_) was selected to further characterize the evaporation flow battery. This section reports the battery electrochemical characterization and the studies regarding the durability of the device's critical components: redox species reactants, the paper core (*i.e.*, paper channels and electrodes), and the evaporation pad. Isolating and studying each component allows the accurate depiction of the battery performance and set-up strategies to further expand the operational lifetime.

### Operation characterization

In order to set up the prototype, 5 mL of the electroactive species previously dissolved in water-based solutions are placed in the reservoirs. Then, the inlet channels are immersed into the solutions. The paper strips absorb the liquid creating a convective flow driven by capillary forces and filling up the system. Once completely full, the evaporation forces occurring at the liquid–air interface of the absorbent pad become predominant over the capillary effect. This is when the flow battery is mainly evaporation-driven and the prototype is ready to be used. In this work, the battery was characterized using a well-known couple of quinone-based organic species, which have been already used as redox couples in power sources.^33,47,52^ This particular chemistry has already shown to successfully undergo anaerobic degradation and non-toxicity in previous studies.^33^ As shown in Fig. 1, both half-cell redox reactions present a proton-dependence; therefore, mixed-media conditions are used as a strategy to increase the electrochemical cell voltage.^53,54^ Ultimately, a solution of 0.1 M *p*-BQ in 0.5 M of oxalic acid is used as the acid catholyte, while 0.1 M H_2_BQS in 1 M of KOH solution composes the alkaline anolyte. The half-cell reaction characterization in a conventional three-electrode electrochemical cell can be found in Fig. S2 (ESI†).

**Fig. 4:**
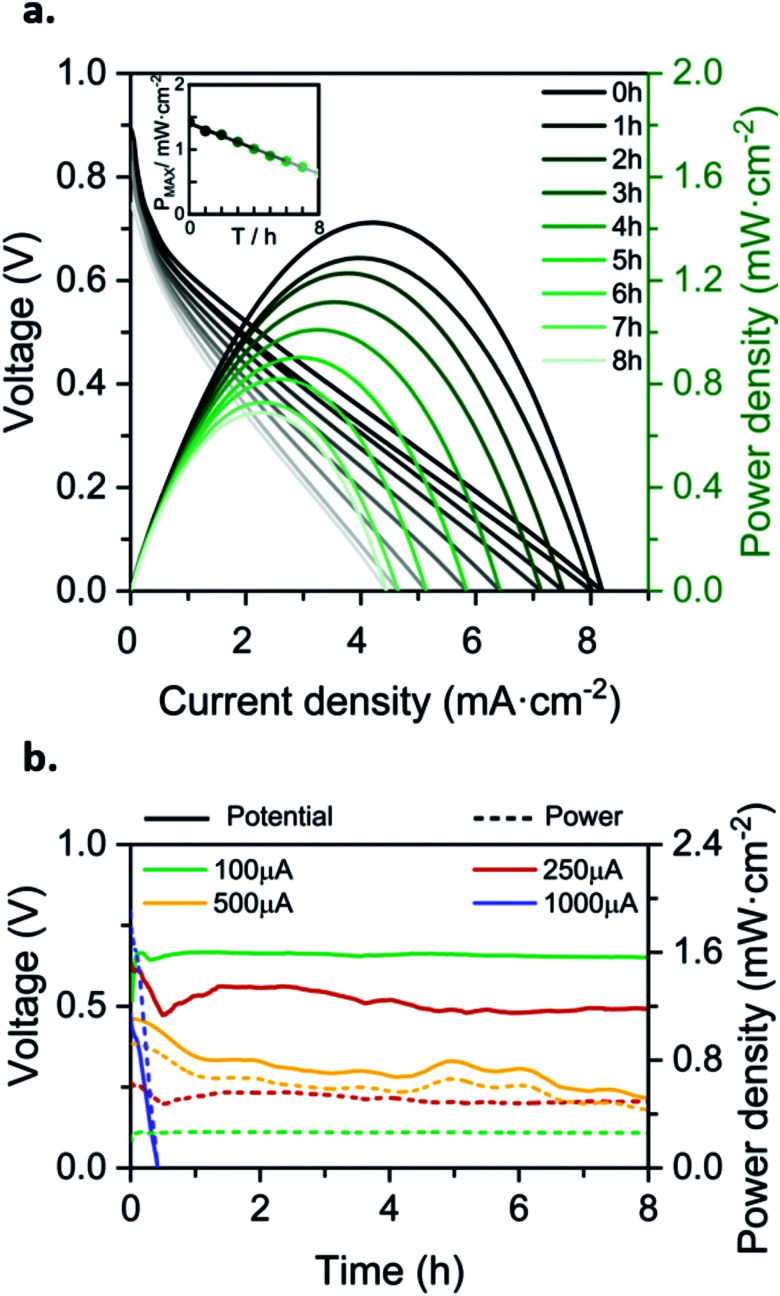
Battery characterization for 8 h, (a) discharge polarization curves of the evaporated driven flow battery, recorded every hour since the start (black line) until the 8th hour (palest line); (b) continuously measured discharge curves at four different discharge currents. Prototype supplied with 0.1 M H2BQS in 1 M KOH as the anolyte and 0.1 M *p*-BQ in 0.5 M C_2_H_2_O_4_ as the catholyte. Average flow rates calculated from eqn (1), and environmental conditions recorded with a commercial sensor: (a) *Q*_evap_ = 239 μL h^−1^. HR% ∈ [54, 57]%, *T* ∈ [19, 21] °C. (b) *I* = 100 μA; *Q*_evap_ = 276 μL h^−1^, HR% ∈ [62, 70]%, *T* ∈ [25, 27] °C; *I* = 250 μA; *Q*_evap_ = 333 μL h^−1^, HR% ∈ [55, 59]%, *T* ∈ [25, 26] °C; *I* = 500 μA; *Q*_evap_ = 260 μL h^−1^, HR% ∈ [58, 63]%, *T* ∈ [23, 24] °C; *I* = 1000 μA; *Q*_evap_ = 351 μL h^−1^, HR% ∈ [55, 63]%, *T* ∈ [26, 28] °C (*P* = 1 atm).

Discrete characterization of the battery was performed for 8h by consecutively recording the polarization curves every hour. The OCV was in all cases between 0.8 and 0.9 V. As depicted in Fig. 4a, the battery yields a maximum power density of 1.42 mW cm^−2^ at time 0 h, and then decreases linearly over time until 0.69 mW cm^−2^ at time 8 h. It is important to notice that this power density is in the same order of magnitude as the maximum power density reported in our previous work (1.88 mW cm^−2^ at 540 μL h^−1^), using the same fluidic system but driven by capillary forces.

Despite showing a decrease in output power during the first 8 h, these results demonstrate that evaporation-driving forces are able to maintain the co-laminarity of the two reactants streams, expanding the operational time of the paper-based battery from minutes to hours. The power density loss causes are further studied in the section below when assessing redox species’ stability.

Next, continuous operation was characterized by measuring battery voltage evolution over time under four discharge currents. Fig. 4b shows the discharge curves obtained in terms of battery voltage and power density. This characterization under continuous operation was used to assess the device efficiency. The faradaic efficiency (*η*_F_) allows evaluation of the electroactive species utilization, quantifying the theoretical charge capacity that is actually being converted into current (see eqn (2)). The energy efficiency (*η*_E_) was also evaluated by comparing the power delivered to the energy that ideally would be delivered (see eqn (3)).

The fluidic battery is able to deliver 100 μA for 8 h without voltage or power decrease, delivering a total of 0.8 mA h of capacity and 13.5% *η*_F_ and 9% *η*_E_ conversion rates. These values are within the average range for the reported paper-based microfluidics electrochemical power sources (1–10% *η*_F_ and 1–5% *η*_E_).^47^ As expected, for higher discharging currents the voltage decreases while efficiencies increase, leading to a 15% decrease for 250 μA (with 2 mA h of capacity, 28% *η*_F_ and 16% *η*_E_) and a 50% for 500 μA (4 mA h of total capacity, 71% *η*_F_ and 24% *η*_E_). The device operating at 500 μA delivered the highest power values (0.2 mW to 0.1 mW) during the whole measurement. In all three cases, the battery was able to maintain a continuous power supply for 8 h. However, when the battery was subjected to 1000 μA working current (*i.e.* 4 mA cm^−2^), the operational working time decays to 30 minutes, yielding a 0.5 mA h capacity, an increase in faradaic efficiency up to 99% *η*_F_ but a decrease in energy efficiency to 12% *η*_E_. Higher current densities provoke a faster depletion of the reactants at the electrodes, thus the galvanic cell evolves from working in ohmic to mass transport loss regions in which the performance decays nonlinearly with current density.^16,55^ Hence, a working condition limitation was found above 500 μA or 2 mA cm^−2^ current density.^47^

### Durability of the device's core

**Fig. 5:**
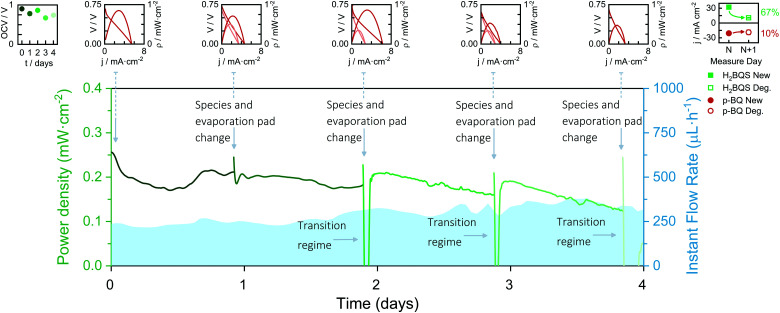
Continuous discharge curve of the evaporation-driven flow battery working at 100 μA for 4 days with daily renewal of redox species solutions and evaporation pad. Insets: Battery OCVs *vs.* time, battery polarization curves before and after evaporation pad and redox species replacement, and anolyte and catholyte degradation study in one day. Average flow rates calculated from eqn (1), and environmental conditions recorded with a commercial sensor: Day 1; *Q*_evap_ = 103 μL h^−1^, HR% ∈ [60, 81]%, *T* ∈ [19, 22] °C; Day 2; *Q*_evap_ = 184 μL h^−1^, HR% ∈ [59, 64]%, *T* ∈ [19, 22] °C; Day 3; *Q*_evap_ = 215 μL h^−1^, HR% ∈ [57, 66]%, *T* ∈ [17, 20] °C; Day 4; *Q*_evap_ = 157 μL h^−1^, HR% ∈ [65, 73]%, *T* ∈ [18, 21] °C (*P* = 1 atm).

To study the durability of the device's paper core (paper channels and electrodes), the battery was set up for four days and subjected to continuous current demand of 100 μA. Every 24 h the anolyte and catholyte reservoirs were refilled with fresh solutions and the evaporation pad was replaced with a new dry one. Moreover, the battery polarization curve was recorded before and after this daily conditioning. Depicted in Fig. 5 are the obtained discharge curves together with the polarization curves of the battery over the days. The battery shows a continuous power delivery of around 200 μW cm^2^ for four days. A transition regime appears when changing the species and the evaporation pad, in which the capillary flow caused by the new evaporation pad replenishment perturbs the battery performance. When analysing the polarization curves, however, it can be observed that the battery performance improves after the daily conditioning during the first three days. This result agrees with the current density peak loss caused by the anodic species degradation, which was obtained from the analysis of the solution in the reservoir every day before replenishment. However, on the fourth day of continuous operation, the battery performance dropped to 0 V. No further improvement was seen from changing the solutions or the evaporation pad, indicating that the device's core operational lifetime was reached.

### Redox species stability

**Fig. 6:**
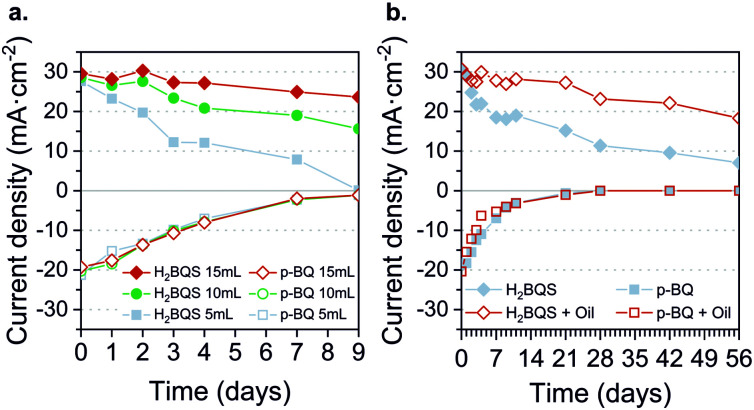
Battery reactant degradation through time. (a) Effect of time storage on different solution volumes. (b) Effect of time storage on 10 mL volume with or without a top protective layer of sunflower oil.

The anolyte and catholyte degradation and its relation with the total loss of performance of the battery were investigated. The redox species were analyzed *via* cyclic voltammetry (CV) for 9 days. Three different volumes (5 mL, 10 mL, and 15 mL) were assessed, in order to study the influence of exposed area on the oxygen *vs.* total solution volume stored. Fig. 6a shows the correlation between the current density peak and storage time for each volume. In the case of the anodic reactant, H_2_BQS, the slope decay was palliated when increasing the total solution volume; the signal loss was 100% for 5 mL solution, 47% for 10 mL, and 20% for 15 mL, showing a direct dependence between oxygen exposure and H_2_BQS spontaneous degradation. Since all samples were stored in the same kind of reservoirs, the contact surface area between the reactive and the air was the same, indicating that the amount of H_2_BQS oxidized by the air was equal for all three samples. Therefore, the different volumes of reagents led to different concentration changes and thus maximum current peaks. With respect to the cathodic species, *p*-BQ, the decay of the current density peak is independent of the storage volume; 94% of the signal loss was measured in all cases. This result agrees with the naturally occurring reduction of *p*-BQ in aqueous media. The reduction product, hydroquinone (HQ), spontaneously polymerizes into large poly-hydroquinone chains, losing its electrochemical activity.^56^ As a consequence of these results, the prototype operating volume was increased to 15 mL.

With the aim of preventing the redox species degradation, deoxygenation with *N*_2(g)_ and storage of the solutions were assessed with CV for 14 days. Fig. S4 (ESI†) shows the current density peaks obtained. This approach demonstrates to successfully prevent the degradation of the H_2_BQS anodic reactant, whereas the *p*-BQ degradation continues regardless of the deoxygenation. Consequently, as a simple and low-cost method to avoid oxygen molecules from reaching the redox species, we added a 2 mm thick layer of vegetable oil on the top of the reactant solutions. The result of species electrochemical response over 56 days (8 weeks) is depicted in Fig. 6b; all stored in the same volume (10 mL) and concentration.

As can be seen, the H_2_BQS current peaks decay less with time when the protective oil layer is present. Quantitatively, only 37% of signal loss was recorded for H_2_BQS with the oil protection, while the bare solution presented a 84% decrease. Regarding *p*-BQ, no stabilization of the redox species was possible, 50% of degradation is reached in 3 days and 100% of degradation in 28 days for both solutions. Confirming that, as previously stated, its degradation comes from the fact of being diluted in a water media and not from being in contact with oxygen from air. The chemical stability of organic redox compounds in aqueous media has been widely studied, due to their interest for redox flow batteries. The quinones containing ketone groups, such as *p*-BQ, generally show instability because their carbon–oxygen double bonds are susceptible to reacting with nucleophiles, such as water, *via* Michael addition.^57,58^

### Optimized continuous operation prototype

**Fig. 7:**
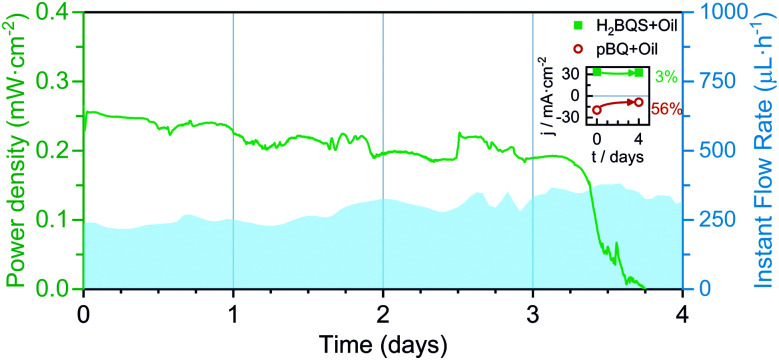
Continuous power density delivered by the evaporation-driven flow battery discharged at 100 μA for 4 days. Also depicted is the flow rate evolution. Inset: 4 day degradation of the anolyte and catholyte solutions. Average flow rates calculated from eqn (1), and environmental conditions recorded with a commercial sensor: Day 1; *Q*_evap_ = 237 μL h^−1^, HR% ∈ [68, 72]%, *T* ∈ [26, 27] °C; Day 2; *Q*_evap_ = 260 μL h^−1^, HR% ∈ [61, 73]%, *T* ∈ [27, 27] °C; Day 3; *Q*_evap_ = 308 μL h^−1^, HR% ∈ [55, 67]%, *T* ∈ [23, 27] °C; Day 4; *Q*_evap_ = 358 μL h^−1^, HR% ∈ [54, 58]%, *T* ∈ [25, 27] °C (*P* = 1 atm for all days).

Finally, an optimized FlowER battery prototype was assembled and tested under a continuous discharge current of 100 μA. In this case, the 2 × *A*_Pad_ configuration was selected and a 2 mm of the protective oil layer was added on the top of the 15 mL solution in the battery reservoirs. The battery yielded a power density between 0.25 and 0.2 mW cm^−1^ with averages of 13.3% *η*_F_ and 7.7% *η*_E_, over 3 days and a half (see Fig. 7). Perturbations on the power delivered can be attributed to changes in the evaporation conditions (*i.e. T*_amb_, *P*, and HR%) that are reflected in the flow rate. The performance drastically drops on the fourth working day. As stated above, the oil protection layer does not prevent the *p*-BQ species from degradation; the catholyte and anolyte solutions were tested and a 56% signal decrease was obtained for *p*-BQ.

Overall, the flow battery operational time was expanded from hours to days. Achieving an operation time that considerably exceeds those demonstrated in the literature for paper-based energy conversion devices (usually in the range of minutes).^47^

After the conducted studies, a few situations that could produce a decrease in the device performance were identified. Depending on the cause, these failure modes can be categorized as effects related to the intrinsic assembly of the battery or to the external events due to the environmental conditions. For example, the evaporation process results in an accumulation of solid compounds in two regions, the evaporation pad and the electrode edges. This effect of saturation of the evaporation area could be mitigated by introducing a washing buffer, whereas the electrode zone sealing could be improved to avoid parasitic evaporation. Regarding working conditions, the prototype has demonstrated good performance under typical greenhouse conditions; however, high working temperatures and/or low relative humidity would increase the flow rate, thus causing early exhaustion of the active materials. In this case, the design should be appropriately scaled to the targeted ambient conditions. Thus providing an adequate volume of active solutions according to the estimated flow rate in the hydrometric chart (Fig. S3, ESI†). Furthermore, extreme ambient conditions, such as heavy rain or flooding, would cause a malfunction. To mitigate the detrimental effect that excess water would have on the paper-based prototype, a breathable impermeable and biodegradable coating (such as natural wax) could be applied onto the paper structure. It is foreseen that these failure modes could be tackled with design optimization and through a tailoring process addressing specific working conditions or requirements. Furthermore, thanks to the modular design, the FlowER battery could be easily modified to enable evaporation pad and/or electrode replacement or accessible refilling of the active solutions, which would also result in an extension of its operational lifespan.

### 2.6 Driving a wireless smart monitoring unit for precision horticulture

With the aim to test a realistic PA application scenario, a Flower Care™ smart monitoring unit was purchased. Flower Care™ is a low-cost device commercialized for horticulture or indoor gardening. The unit encompasses four sensors for measuring essential PA parameters. In particular, the unit can concurrently monitor the soil conductivity, soil humidity, air temperature, and light exposure of a nearby plant. Flower Care™ runs the Bluetooth Low Power (BLE) protocol for transferring the data measured on a smartphone (see Fig. S5–S7, ESI†). Normally, a primary 3 V cell coin battery (*i.e.*, lithium battery model CR2032) powers the device. As a first prerequisite to drive the sensor's internal circuitry using the FlowER battery, we need to amplify the voltage generated (initially inferior to 1 V, see for instance Fig. 4). A common strategy used in the literature to increase the output voltage of paper power sources is cell stacking; different studies have reported stacking designs and their effects on the cell output.^59–63^ In this work, however, a voltage boost converter (VBC) integrated circuit was used to amplify the operating voltage up to 3.1 V (see additional details in ESI†). We favoured this option because the commercial Flower Care™ sensing unit is not an eco-friendly sensing unit; namely, it is made of conventional electronic components (*e.g.*, printed circuit board, integrated circuits, *etc.*). Consequently, the additional use of the VBC did not appear critical for demonstration purposes at this stage of development.

In addition, it turns out that the communication modes of the BLE protocol frequently imposed current peaks up to 5–6 mA (*e.g.*, see Fig. S6 and S7, ESI†). This means net power consumption peaks up to ≈18 mW. Comparatively, the maximum net power generated by the FlowER battery did not exceed 0.4 mW (see maximum power points in Fig. 4). To overcome this difficulty, a 6.8 mF super-capacitor was selected as a refillable energy reservoir capable of absorbing any punctual current/power peaks that could not come directly from our evaporation-driven battery.

**Fig. 8:**
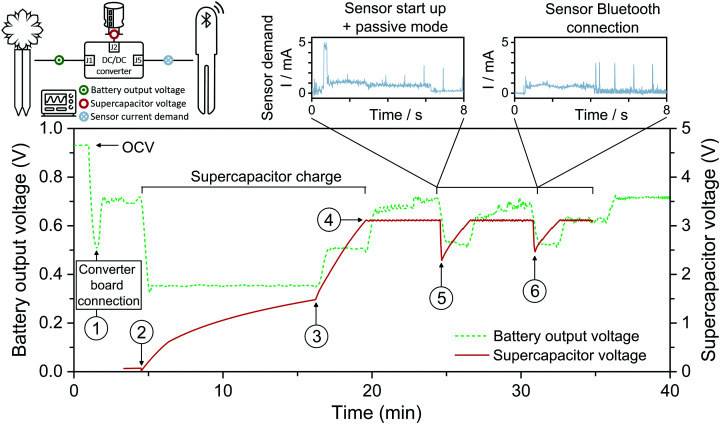
Proof of concept of the evaporation driven flow battery demonstrating the capability of powering the start-up, passive mode and Bluetooth connection of a horticulture caring device.

First, the capability of the FlowER battery to fully charge the super-capacitor fully discharged was verified. As soon as the FlowER battery with the VBC was connected, the voltage generated abruptly dropped from its initial OCV value to 0.5 V (see ① in Fig. 8). The battery output dropped further down to ≈0.3 V when the VBC started to charge the super-capacitor (see ②). The full charging process took about 15 min, with two distinct phases. Indeed, the VBC needed to overcome a cold-start phase before it could normally charge the capacitor. During the first slower charging rate phase (see ② and ③), the current demand was higher. Comparatively, the charging rate became much higher during the last 3 minutes of the process (see ③ and ④). In the course of this second phase, the VBC's internal circuitry could exploit its advanced non-linear switch-based technique to optimize the voltage across the storage element. As a direct consequence, the current demand decreased and the battery voltage re-increased to 0.5 V. At the end of the charging process (*i.e.*, when the super-capacitor voltage reached 3.1 V), the current consumption dropped to the nano-microamps range and the FlowER battery voltage returned swiftly to 0.7 V (see ④). After the full charge of the super-capacitor, the Flower Care™ sensor was turned on. At this moment, the capacitor voltage suddenly dropped from 3.1 to 2.3 V (see ⑤). This was due to the start-up sequence of Flower Care™ (see Fig. S5, ESI†). Once activated, the sensor entered into the sleep mode and ran advertising events every 1 s (see Fig. S6, ESI†). These events also include three current peaks reaching 6 mA, but their duration was very short (<1 ms). Despite their presence, the battery proved to be powerful enough to recharge the capacitor back to 3.1 V in less than 2 min. The sensor can thereby stay activated and run the advertising mode as long as needed. To transfer data, the Flower Care™ was connected to the smartphone. A second sudden drop in the capacitor voltage occurred (see ⑥). Using this scenario, Fig. 8 shows that the FlowER feasibility battery could successfully recharge the capacitor and maintain wireless data transmission to a smartphone. Despite the fact that this first proof-of-concept is based on the use of additional external electronic components (*i.e.*, the VBC and a super capacitor), it clearly demonstrates that the FlowER battery is already capable of generating enough electrical power to drive off-the-shelf electronic components useful for PA applications. Thanks to the modularity of its design, the FlowER battery hence appears as an ideal candidate to supply power to novel approaches in green electronics that are being developed for the smart agriculture sector, also pursuing biodegradability as plausible electronics end-of-life.^64–66^

### 2.7 Aerobic biodegradability and phytotoxicity tests

The FlowER battery is intended as a sustainable power supply for PA applications. For this reason, it is compelling to demonstrate that the prototype end-of-life is completely aligned with this purpose. The FlowER battery green casing structure is made of COMPOST3D, a commercially available material certified as biodegradable under OK Compost standard (OK Compost HOME, TÜV Austria). Hence, the tests reported in this section analyse the evaporation pad as a representative portion of the paper structure which, furthermore, ends up having the highest accumulation of redox species and electrolyte salts. Biodegradation pathways for standard organic molecules as cellulose or simple aromatic compounds, like the battery components, are well stablished in the general literature related to composting and bioremediation.^67–71^ Both natural environment (soil) and composting matrices are complex ecosystems with a rich microbiome (including meso and thermophilic bacteria and fungi). Therein, molecules are first hydrolyzed by extracellular enzymes to simpler molecules and later fully mineralized to CO_2_ and H_2_O by the involved microorganisms.

**Fig. 9:**
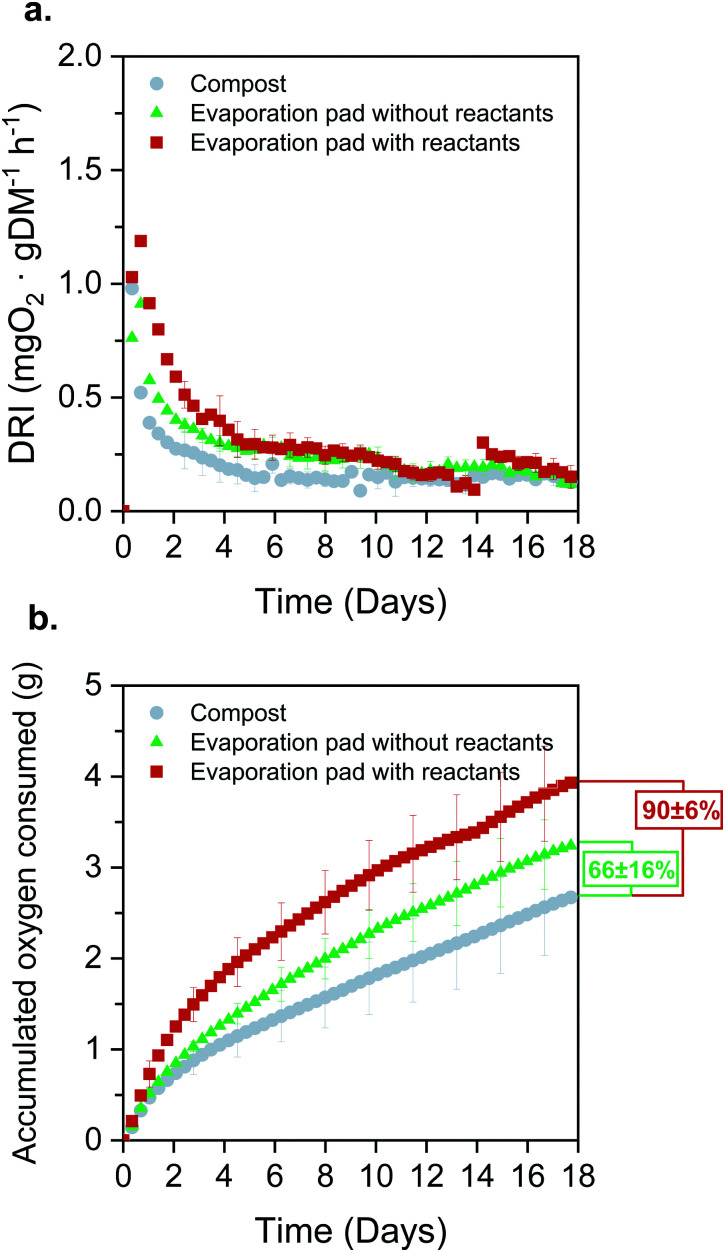
Aerobic biodegradability assessment of the FlowER battery, (*N* = 3). (a) Dynamic respiration index (DRI) of evaporation pads with and without the reaction products accumulated over 3 days and compost used as a blank; and (b) total oxygen consumed by the three samples and level of biodegradation achieved at day 18.

First, biodegradability was assessed under controlled aerobic conditions and following a procedure adapted from the standard EN 13432.^72^ Three different types of samples were analysed: (i) compost alone (used as control); (ii) compost with un-used evaporation pads (*i.e.*, cellulose without reactants); (iii) compost with used evaporation pads (*i.e.*, cellulose with reaction products accumulated for 3 days). The dynamic respiration index (DRI) was computed as an indicator of the microbial activity evolution through the degradation process using eqn (4).^73,74^ The high initial DRI values (Fig. 9a) indicate a rapid microbial growth derived from the presence of easily biodegradable carbon. This behaviour is even sharper in the case of the evaporation pad with reaction products. Moreover, the dynamics up to day 12 support this trend, confirming that the samples are acting as a substrate and being consumed by the consortium of microorganisms present in the compost. From day 15 onwards, the DRI curves flatten and there are no remarkable differences in the microorganisms’ activity for the different samples.

Fig. 9b depicts the accumulated oxygen uptake (eqn (5)) by the three samples over 18 consecutive days. The total oxygen consumed was 2.75 ± 0.54 g for the compost, 3.30 ± 0.38 g for the evaporation pads without products, and 4.02 ± 0.6 g for the evaporation pads with accumulated products. Total oxygen consumed by the microorganisms during the biodegradation process allows for the calculation of the percentage of biodegradation as compared to the theoretical oxygen demand (eqn (6)). Standard EN 13432 requires 90% of biodegradation in less than 6 months to consider a material as biodegradable. The levels of biodegradation reached in only 18 days were 66 ± 16% and 90 ± 6% for the evaporation pads without and with reactants, respectively. It is noteworthy that the oxygen uptake rate of the used evaporation pads was considerably higher than that of the unused. This may be due to physico-chemical changes in the cellulose structure during the battery lifespan or to the presence of the reaction products, both factors favouring microbial degradation of the evaporation pads. This effect was confirmed when the reactors were opened at day 18 and non-recognizable parts of the sorbents were found in evaporation pads with reactants while small fragments from un-used pads were observed in the final compost.

After the aerobic biodegradation assay, seed germination tests were carried out in order to assess the toxicity of the three generated composts. Germination index (GI), relative root elongation (RRE), and germination percentage of all composts are shown in Table 1. GI values over 100% are considered to have a positive effect on germination and root elongation whereas composts with GI values higher than 80% assure the absence of phytotoxic substances.^75^ As observed, compost control has a positive effect both in IG and RRE. Regarding compost from aerobic biodegradation of evaporation pads with reaction products, RRE does not show significant differences from that of compost control, whereas the percentage of germination is slightly lower. In any case, the GI is higher than 100% ensuring its beneficial use. However, when the same chemical species used in the battery were added directly to a compost control but not subjected to a biodegradation process, a significant negative effect is observed. This effect is especially harmful in RRE, inhibiting radicle growth, which is a more sensitive indicator of toxicity than the percentage of germination.^76^ The evaporation pad without species (cellulose) used as a control affected more than expected the germination indices. We assume this may be due to the incomplete degradation of cellulose during the test. Instead, as mentioned above, the alterations of the cellulose structure during the battery operation and the presence of the reaction products seem to speed up evaporation pad degradation and lead to a more stable, harmless final material.

**Table 1:** Germination test for compost generated from biodegradation assessments of evaporation pads of the FlowER battery: with and without products in comparison with a compost control and compost with pure reactants added directly. Pictures show the differences between the representative samples analysed. The results have been statistically analysed using the Holm–Sidack pairwise multiple comparison procedure; hence the compost samples have been paired under different letters. Those samples paired under the same letter showed non-significant differences (*p* < 0.05) (Holm–Sidack Test, Sigmaplot 11)

Compost generated from the biodegradability assessment of	Germination index (%)	Percentage of germination (%)	Relative root elongation (%)
Control compost	195 ± 75^a^	100 ± 20^a^	195 ± 83^a^
Evaporation pads with products	77 ± 44^c^	81 ± 12^b^	95 ± 61^b^
Evaporation pads with reaction products	147 ± 46^b^	86 ± 15^b^	171 ± 65^a^
Control compost with pure reactants	7 ± 5^d^	52 ± 4^c^	13 ± 6^c^
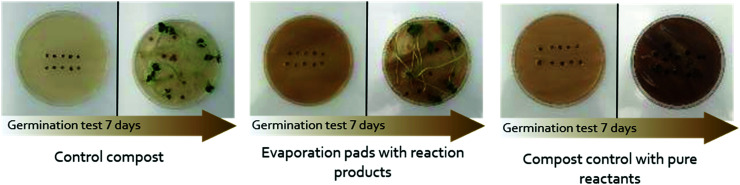			

Altogether, the results reported above confirm the FlowER battery's capability to be aerobically biodegraded according to standardized tests. Furthermore, a nutritious non-toxic compost was obtained from the biodegradation test, ensuring the harmlessness of the biotic degradation products.

## 3. Experimental section

### Chemicals

The redox species used for setting up the battery were *p*-benzoquinone (*p*-BQ) and hydroquinone sulfonic acid potassium salt (H_2_BQS), with oxalic acid and potassium hydroxide (KOH) as electrolytes. All of them were purchased from Sigma-Aldrich (Sigma Aldrich, St Louis, Missouri, USA) and used as received, as well as the blue colorant used for the evaporation flow rate characterization, erioglaucine disodium salt. All solutions were prepared in deionized water; 0.1 M *p*-BQ in 0.5 M C_2_H_2_O_4_ and 0.1 M H_2_BQS in 1 M KOH were used as catholyte and anolyte, respectively.

### Device fabrication

The device's paper-based fluidic system was designed using a CAD program (CorelDRAW, Corel, Ottawa, ON, Canada). The paper structure was fabricated as follows: due to its low fluidic resistance, glass fiber (Standard 14 355 μm thick) is chosen as inlet channels; whereas cellulose (Whatman 1180 μm thick) is used to fabricate the outlet channels, fluidic connector and absorbent/evaporation pad. Whatman 1 material is a well-known cellulosic filter paper, typically used in microfluidics as an absorbent pad, since it creates a homogeneous capillary pressure. This material seemed also adequate as the evaporation pad because it is one of the thinnest commercially available cellulosic filters, a property that allows the reduction of the absorbent volume *versus* its superficial area, resulting in an enhancement of the total liquid–air interface. Both materials were purchased from GE Healthcare, Pittsburgh, PA, USA. These paper-like materials were cut using a CO_2_ laser cutter (Mini 24, Epilog Laser, Golden, CO, USA). Porous carbon electrodes were cut to size (10 × 5 mm) from sheets of Toray carbon paper (TGPH-090 E-TEK, 280 μm). For the purpose of this work, the porous carbon electrodes were thermally pre-treated to confer them with hydrophilic behaviour.

A compostable and 3D printable filament, COMPOST3D, was used for building the mechanical structure (B4Plastics, IQ Parklaan 2A, 3650 Dilsen-Stokkem, Belgium), by 3D-printing a custom-made model, designed using an open-source software, Blender (Blender Foundation, Stichting Blender Foundation, Buikslotermeerplein 161, 1025 ET Amsterdam, the Netherlands). The different parts of the prototype were manually assembled with a water-based bioadhesive, BioTAK S100, a double-sided PSA formulated to include a high content of renewable materials (BioTAK, Sustainable Adhesive Products B.V., Venkelbaan 82, NL-2908 KE Capelle aan den IJssel, The Netherlands).

### Flow rate characterization

The experimental evaporation flow rate over time was characterized by measuring the decrease of the liquid height in device trays. To enhance image contrast, in these experiments, 100 mM solution of euroglaucine disodium salt dye was used to simulate and visualize the anolyte and catholyte flow. The images were recorded with a camera (C920, Logitech, Fremont, CA, USA) controlled by time-lapse software (Sky Studio Pro, free licensed), which saved images at a rate of 1 frame every 20 min. The flow rates were calculated from the captured images by measuring the height decrease in the trays over time. The images were analysed by using ImageJ software (US National Institutes of Health, Bethesda, Maryland, USA).

The theoretical evaporation flow rate over time was characterized through the environmental conditions of humidity and temperature, recorded with a sensor (ALPS Sensor IoT Network Smart Module, Alps Electric Co., Ltd, Osaki, 04, JP).

### Electrochemical studies

The effect of storage time on redox species degradation was characterized *via* cyclic voltammetry. In the case of different storage volumes and deoxygenation studies, the measurements were performed in a three-electrode electrochemical cell. Glassy carbon electrode (0.071 cm^2^), platinum electrode, and Ag/AgCl electrode (CH Instruments Inc., TX, USA) were used as the working, counter and reference electrodes, respectively. The effect of the sunflower oil protection layer was assessed on carbon SPEs (screen-printed electrodes), which use Ag/AgCl as the reference electrode (fabricated by the authors).

The polarization and power curves for battery characterization were generated using linear sweep voltammetry technique, from premeasured OCV to 0 V at a scan rate of 20 mV s^−1^. The discharging curves were generated using chronopotentiometry technique by holding the battery at different discharging currents.

All electrochemical measurements were recorded with a DropSens μStat400 bipotentiostat/galvanostat and DropView 8400 Software (DropSens S.L., Asturias, Spain), at a scan rate of 20 mV s^−1^.

### Faradaic and energy efficiencies

The faradaic efficiency allows the evaluation of the redox species utilization, by comparing the total theoretical charge capacity with the one experimentally harvested. It can be computed using the following expression:2
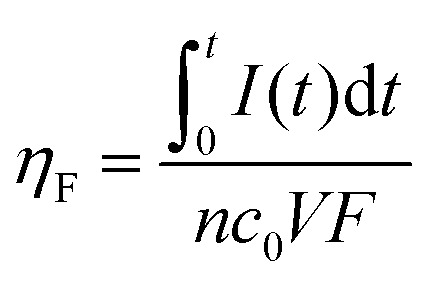
where *I*(*t*) is the current recorded or demanded during the measure, *t* is the measurement time, *n* is the number of electrons per mole of reactive specie, *c*_0_ stands for the bulk species concentration, *V* is the total volume that has gone through the electrodes and *F* is the Faraday constant.

On the other hand, the energy efficiency compares the energy that the battery could theoretically deliver with the power actually delivered under continuous operation. It can be expressed as follows:3
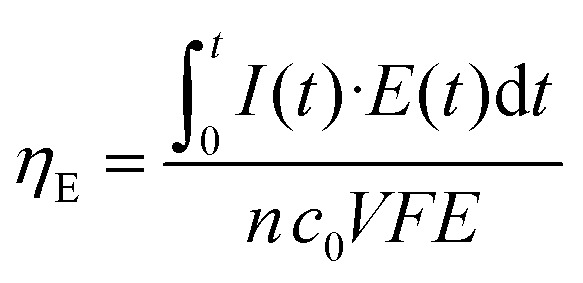
where *E*(*t*) is the operating voltage and *E* is the theoretical Nernst cell voltage.

### Flower Care™ sensor and voltage boost converter

A Flower Care™ (HHCC-Xiaomi, Beijing, China) wireless plant monitoring unit was purchased from an ecommerce platform. The accompanying app was installed on a smartphone running Android 11. The sensor was preliminary linked to the app before running experiments.

To monitor the voltage variations of the FlowER battery under load, a portable potentiostat/galvanostat was used (Dropsens μStat400, DropSens S.L., Oviedo, Spain) coupled to the Dropview 8400 software installed on a laptop.

To amplify the initial voltage generated by the FlowER battery, the evaluation module of an ultra-low-power DC-DC boost converter (BQ25504-EVM, Texas Instruments, Dallas, USA) was used in association with a 6.8 mF super-capacitor (BZ05FB682ZSB, AVX). The BQ25504-EVM is equipped with three terminal blocks: the input source (J1), the charger output (J5), and the battery connection (J2). The FlowER battery was connected to J1 after the establishment and stabilization of the evaporation phase (*i.e.*, the evaporation effect became dominant over the capillary effect so that the battery was predominantly evaporation-driven). The Flower Care™ sensor and the super-capacitor were connected to J5 and J2, respectively. The functions available on the evaluation board (*e.g.*, maximum power tracking point, under-voltage settings, *etc.*) were used as provided by the manufacturer.

Electrical signals with a duration ranging from a few milliseconds to several tens of seconds were measured using a digital oscilloscope (DSOX2002A, Agilent Technologies, Santa Clara, USA and Waverunner 44Xi, LeCroy, Chestnut Ridge, USA) coupled to a standard probe (P2200, Tektronix). Built-in high-resolution and/or fine modes were used to filter the background noise and facilitate the visualization of the overall signal shapes.

Other electrical signals lasting longer than a few tens of seconds were recorded using a digital multimeter 6.5 digit resolution (34401A, Hewlett Packard, Palo Alto, USA). The multimeter was connected to a computer using a GPIB-USB-HS Card (National Instruments, Austin, USA). A block diagram-based interface in LabVIEW (National Instruments) was developed to automate the acquisition of the data.

### Aerobic biodegradability and phytotoxicity assessment

Aerobic biodegradability tests were performed in 0.5 L self-made PVC cylindrical reactors (13 cm high × 7 cm diameter) with forced air inlet (at 20.9% O_2_) and outlet. A constant flow rate was controlled with a mass flow meter (Mass-Stream D-6311, Bronkhorst, NL) at 15 ml min^−1^.^74^ The incubator (Memmert IF 160, Schwabach, Germany) temperature was set at 58.9 °C to favour the growth of thermophilic microorganisms. Compost moisture content was initially adjusted to 54% and maintained by constant water vapour-saturation of the inlet air. Three samples were analysed: 60 g of compost from industrial plant (Consorci per a la Gestió dels Residus del Vallès Oriental, Barcelona, Spain) were used as a control, 60 g of compost plus three un-used evaporation pads (0.49 g of cellulose) and 60 g of compost plus three used evaporation pads (1.30 ± 0.05 g of cellulose and reaction products accumulated for 3 days). The quantities were set based on proportions proposed by Ruggero *et al.* (2019), slightly reduced due to sample quantity limitations.^77^

The oxygen content at the outlet air flow was monitored using electrochemical O_2_–A_2_ oxygen sensors (Alphasense, UK). The Dynamic Respiration Index (DRI) was determined following UNI 11184: 2016,4
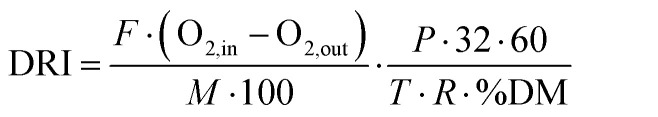
where DRI (mg O_2_ h^−1^ g^−1^ DM); *F*, the air flow rate into the reactor (L min^−1^); O_2,in_, the percentage of oxygen at the inlet air flow (%, mol O_2_ mol^−1^ O_2_), in this experiment was set at 20.9; O_2,out_, the oxygen concentration at the outlet air flow (%, mol O_2_ mol O_2_^−1^); *M*, the total mass inside the reactor (g); *P*, atmospheric pressure (atm); 32 oxygen molecular weight (%, mol O_2_ mol^−1^ O_2_); 60, conversion factor from minutes to hours; *T*, ambient temperature (K); *R*, ideal gas constant (0.08206 L atm mol^−1^ K^−1^); and %DM, fraction of dry matter (g DM g^−1^).^78^

The accumulated oxygen consumed (g O_2_) depicted in Fig. 9b is obtained by the integration of DRI and then multiplying by the sample dry mass (g);5
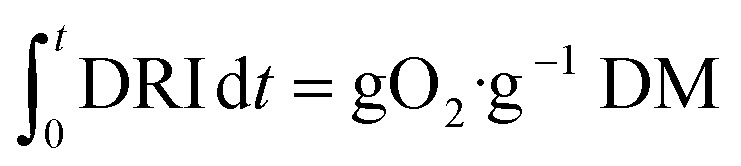
Finally, the biodegradation level (%) was obtained as follows:6
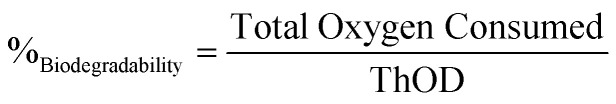
The total oxygen consumed (g O_2_) was calculated by the accumulated oxygen uptake of the sample in 18 days while ThOD is the theoretical oxygen demand (g O_2_). ^79^ The calculated samples’ ThOD are 0.576 g for the evaporation pads without reaction products and 1.408 g for the used evaporation pads.

A germination test using radish seeds (*Raphanus sativus*) was performed to evaluate compost phytotoxicity.^80^ A compost extract (10 mL) in a ratio of 10 : 1 (water volume, in ml, to dry weight, in g) was used to evaluate the germination of 10 radish seeds plated in a Petri dish in triplicate. Germinated seeds and root length were measured after five days in a lighted area at room temperature. Seed germination percentage and relative root elongation (RRE) were calculated and expressed as a percentage about the results of the control using distilled water. Germination Index (GI) was calculated considering both germination percentage and RRE as described by Komilis and Tziouvaras (2009).^75^

## 4. Conclusions

Technological solutions are currently being used to tackle the challenges of digitalized societies and ensure a resilient food system. At the same time, waste electrical and electronic equipment is now the fastest growing waste stream worldwide, being accumulated in landfills and generating considerable environmental and human health risks. In this context, the FlowER battery presented herein aims to evince that it is possible to redefine technological priorities. By placing sustainability as a core priority, feasible and efficient solutions can be created while having a neutral or positive environmental impact. Specifically, conceived and eco-designed to follow the lifecycle of agriculture procedures, the FlowER battery mimics plant mechanisms of fluid transport to passively move reactants through a paper-based fluidic structure. This plant-inspired operation principle enables the creation of a flow-through configuration cell for energy harvesting. Due to transpiration phenomena, co-laminar flow is maintained and reactants are refreshed on the electrode surface. Thus, the need for external pumps is bypassed, while overcoming the operation time limitations of capillary-based flow cells. Parameters affecting the evaporation flow rate and battery performance have been studied, leading to an energy autonomy of up to four days with the present configuration. Operational time was found to be constrained to the particular selected quinone redox couple degradation; thus it could be expanded by using more stable redox chemistries. The prototype practicality has been tested by powering a wireless plant monitoring system, providing enough energy for the start-up, passive monitoring, and Bluetooth communication sequences. Ultimately, the results obtained in aerobic biodegradation and germination tests show that the depleted FlowER battery could be safely disposed of or composted as agricultural waste, resembling the way a plant comes back to nature at the end of its lifecycle.

## Author contributions

Marina Navarro-Segarra: conceptualization, validation, investigation, data curation, writing – original draft, writing – reviewing and editing, visualization, and project administration. Carles Tortosa: conceptualization, validation, investigation, data curation, writing – original draft, and visualization. Carlos Ruíz Díez: validation, investigation, data curation, and writing – original draft. Denis Desmaële: validation, investigation, data curation, and writing – original draft. Teresa Gea: validation, resources, writing – reviewing and editing, and supervision. Raquel Barrena: validation, investigation, data curation, writing – original draft, and supervision. Neus Sabaté: conceptualization, validation, resources, and funding acquisition. Juan Pablo Esquivel: conceptualization, methodology, validation, resources, writing – reviewing and editing, supervision, project administration, and funding acquisition.

## Conflicts of interest

There are no conflicts to declare.

## Supplementary Material

EE-015-D2EE00597B-s001
