# Supplementary material for: A plant-like battery: a biodegradable power source ecodesigned for precision agriculture
Source: Energy Environ Sci. 2022 May 30;15(7):2900–15. doi: 10.1039/d2ee00597b (PMC9277620; doi:10.1039/d2ee00597b)
Supplement: EE-015-D2EE00597B-s001 [file EE-015-D2EE00597B-s001.pdf]

Electronic Supplementary Material (ESI) for Energy & Environmental Science.
This journal is © The Royal Society of Chemistry 2022

Supporting Information

A plant-like battery: biodegradable power source ecodesigned for precision agriculture

Marina Navarro-Segarra,[a] *Carles Tortosa,*[a] *Carlos Ruiz-Díez,*[a] *Denis Desmaële,*[a] *Teresa Gea,*[b] *Raquel Barrena,*[b] *Neus Sabaté,*[a,c] *Juan Pablo Esquivel* [*a,d,e]

a. Instituto de Microelectrónica de Barcelona, IMB-CNM (CSIC), C/ dels Til·lers sn, Campus UAB, 08193 Bellaterra Barcelona, Spain

b. Universitat Autònoma de Barcelona (UAB), 08193 Bellaterra Barcelona, Spain.

c. Catalan Institution for Research and Advanced Studies (ICREA), Passeig Lluís Companys 23, 08010 Barcelona, Spain

d. BCMaterials, Basque Centre for Materials, Applications and Nanostructures, UPV/EHU Science Park, 48940 Leioa, Spain

e. IKERBASQUE, Basque Foundation for Science, 48009 Bilbao, Spain

Supplementary material

Device components

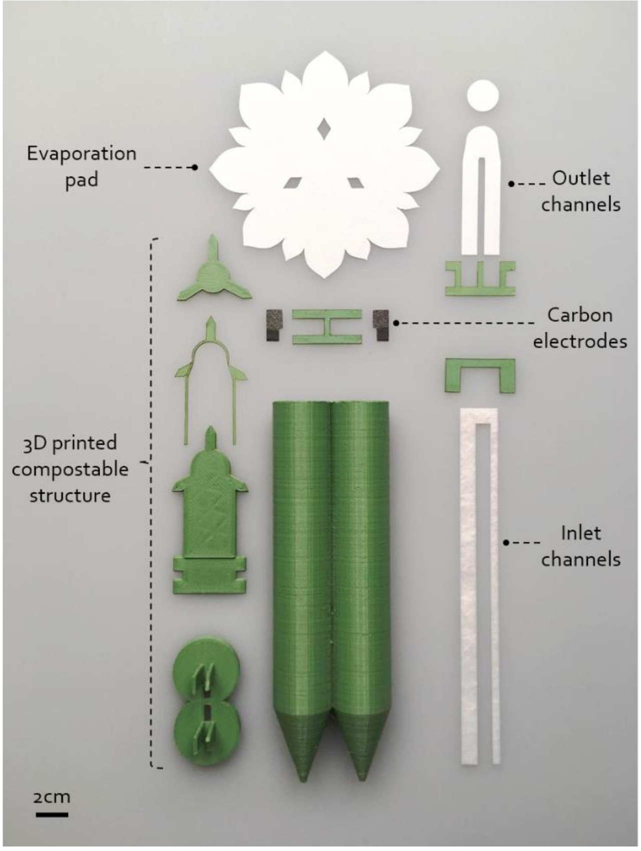

Figure S1. Exploded view of the *FlowER* battery components. The battery comprises a fluidic system composed by a paper-based backbone, two carbon electrodes, and a 3D printed compostable structure.

Half-cell reactions

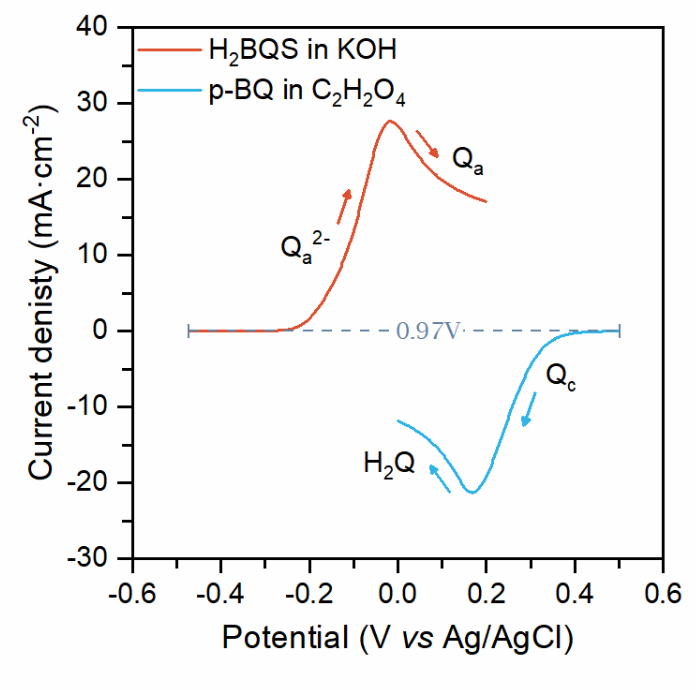

Figure S2. Glassy carbon voltammograms for the negative (0.1M H_2BQS in KOH 1M) and positive (0.1M p-BQ in $C_2H_2O_4$ 0.5M) half-cells; the measured OCPs are -0.47 V and 0.5 V versus Ag/AgCl electrode, respectively.

Derivation of the evaporation rate equation

The evaporation rate equation for a liquid in evaporation was derived using the continuity equation at the interface of the two phases, where the process is occurring. For a control volume within the evaporation interface is given by:

$$Q = \oint_S \rho_v \vec{v} \cdot \hat{n}\, dS$$

where Q is the evaporation rate [g·s^{-1}], S is the surface of the control volume [m^2], ρ_v is the gaseous phase density [g·m^{-3}], $\vec{v}$ is the particles velocity at S [m·s^{-1}], and $\hat{n}$ is the normal vector of the surface S [adim].

While evaporating, there's only flow on the top face of the cylinder, of area *A*, leaving the integral as follows:

$$Q = \rho_v v \oint_S dS = \rho_v v A$$

Since mass transport from the interface surface until bulk region is not driven neither by migration nor convection (assuming forced convection from an external air-flow is null, given laboratory conditions), only diffusion is left as the main cause. Thus, diffusion flow J from a substance in dissolution is given by:

$$J = -D \frac{\Delta \phi}{\Delta x}$$

where *D* is the diffusion coefficient of the solute [m^2·s], and $\Delta\phi$ is the concentration gradient of the solute between two points in space $\left(\frac{\phi_f - \phi_0}{x_f - x_0}\right)$ [mol·m^{-4}].

Applying the diffusion flow equation to this situation, the concentration gradient between two opposite spots of the control volume will be determined by the humidity of the air at each point: liquid-gas interface is saturated at 100% of humidity (H_{sat}), while at bulk region there will be the room humidity (H_r). This way, the following expression is obtained:

$$J = -D \frac{H_r - H_{sat}}{\Delta x}$$

Since the room humidity can be expressed in terms of the relative humidity (in percentage) times the saturation humidity, the previous expression rewrites as follows:

$$J = -D \frac{H_{sat} \cdot HR\% - H_{sat}}{\Delta x} = D \cdot H_{sat} \frac{(1 - HR\%)}{\Delta x}$$

where D is the diffusion coefficient of the water vapour [m^2·s] (dependent on T at P=*cnst.*), H_{sat} is the saturation humidity [g·m^{-3}] (dependent on T at P=*cnst.*), $HR\%$ is the relative humidity [adim] (in percentage), and Δx is the distance between the two studied points.

Since *J* is a flow of mass over area and time $\left[\frac{mol}{m^2 \cdot s}\right]$, when it's multiplied by a certain area (scalar product $\vec{J} \cdot \hat{n}A$) the result is the amount of matter going through that area per unit of time, being equivalent to the situation given at the top part of the control cylinder (in which case the vectors are simplified to a one-dimensional expression, since both the diffusion flow and the surface are parallel).

By the continuity equation, it's known that this amount of matter is the same that is being evaporated. Thus, the following relation is obtained:

$$Q = \rho_v v A = J \cdot A$$

Meaning so that the evaporation flow rate [g · s^{-1}] will be determined by:

$$Q = ADH_{sat}\frac{(1 - HR\%)}{\Delta x}$$

For the purpose of this work D has been approximated to 25.6 $\cdot 10^{-6}$ $m^2 \cdot s^{-1}$, its value at T = 20ºC. and Δx is set as 10^{-6} m to normalize the orders of magnitude of experimental and theoretical flow rates.

Hygrometric Chart

Since the *FlowER* battery is driven by evaporation, its performance is directly dependant on the surrounding ambient conditions. For this reason, a hygrometric chart has been constructed as a tool to predict the battery performance under different conditions. The chart, shown in Fig. S3, represents the effect of the two most influential physical parameters (the temperature and the relative humidity) on our variables of interest (the theoretical flow rate and the output power). The temperature range considered for this study was 5-35 ºC, while the relative humidity included the full range from 0% to 100%. Temperatures below 5 ºC were discarded since they would compromise the flow of the active solution, due to water solvent freezing. The theoretical flow rate was computed by the model developed in Section 2.4. and the theoretical power density peak can be calculated by adapting Faraday's law as follows:

$$P_{peak,theo.} = n\, c_0\, Q\, FE \qquad \text{(S1)}$$

Where n is the number of electrons per mole of reactive species, c_0 stands for the bulk species concentration, Q is the theoretical flow rate, F is the Faraday constant and E is the theoretical Nernst cell voltage. Finally, a correction factor of 0.251 was applied to take into account all the losses affecting the cell performance. The correction factor is computed as the ratio between the power density peak delivered by the battery at time 0h (shown in Fig. 4a) and the theoretical power density peak calculated with eq. S1 for the corresponding working flow rate (Q_{evap}= 239 µL/h).

As depicted in Fig. S3, the *FlowER* behaviour can be predicted in a wide range of temperature and relative humidity conditions. The highlighted areas stand for laboratory conditions recorded over the course of this work (orange) and greenhouse optimal conditions (green).[2] Recalling that the *FlowER* battery has been conceived to power precision agriculture applications it is interesting to analyse the battery performance within a greenhouse environment. For instance, the theoretical power output for the worst-case scenario (HR% = 80% and T = 18 ºC) would be 2.2 mW·cm^{-2}, which translates to 0.6 mW·cm^{-2} after applying the efficiency factor. Conversely, in the warmest (T = 25ºC) and less humid (HR% = 70%) greenhouse environment, the battery would yield a corrected power density of 1.25 mW ·cm^{-2}.

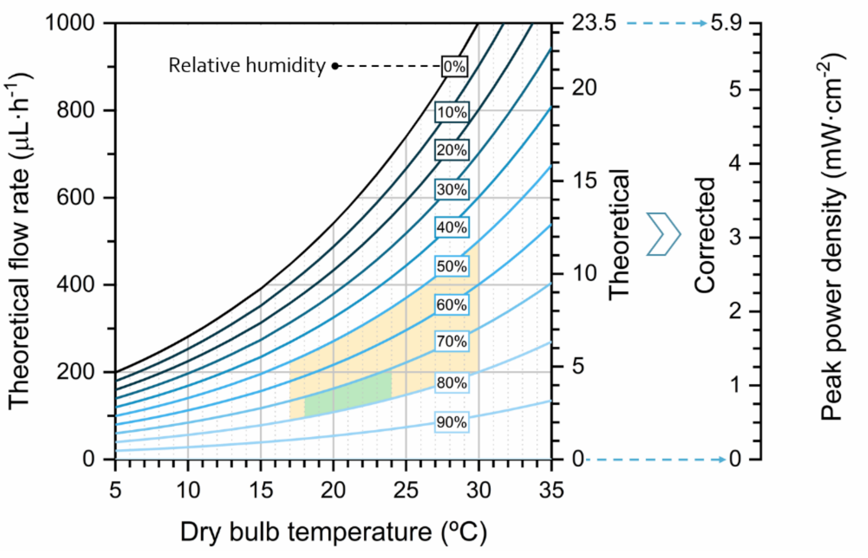

Figure S3. Hygrometric chart to predict the *FlowER* battery performance under different ambient conditions. A correction factor has been applied to take into account battery efficiency. Highlighted areas represent the windows of different ambient conditions: orange area stands for laboratory conditions recorded over the course of this work; green area covers optimal greenhouses conditions.

Species storage: Oxygenation state

To optimize the storage of the redox species, two experiments were set-up: deoxygenation with $N_{2\,(g)}$ and covering of the reactants surface with a hydrophobic material (in this case, sunflower oil). Both were designed to avoid oxygen molecules from reaching the redox species, thus keeping the anodic components in the original state and, *a priori*, showing no difference with the cathodic ones. Here is presented the first one, as the second one is already presented in the main text.

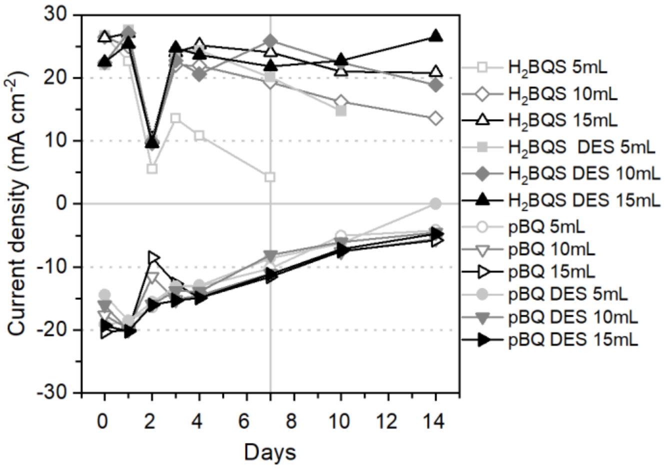

Figure S4. Current density peaks of the battery redox couple for various volumes and oxygenation states (redox species concentrations: 100mM). Hydroquinone Sulfonic Acid (H_2BQS) shows a bigger difference between its samples than p-Benzoquinone, as predicted.

Electronics

Characterization of the Flower Care™ monitoring unit

To get insights into the functioning of the commercial Flower Care™ unit and get an estimate of its power budget, we inserted a 10-Ω resistor (1/4W, 10% tolerance) between 3V coin cell battery (CR2032 format) and the device terminal connectors. We then measured the voltage drop across the resistor using an oscilloscope probe (P2220, Tektronix). Using such a shunt resistor is a simple common reported solution[1]. We selected a 10-Ω value because it is small enough so that it does not affect the existing circuitry; but it is large enough so that we could measure a voltage that with enough precision.

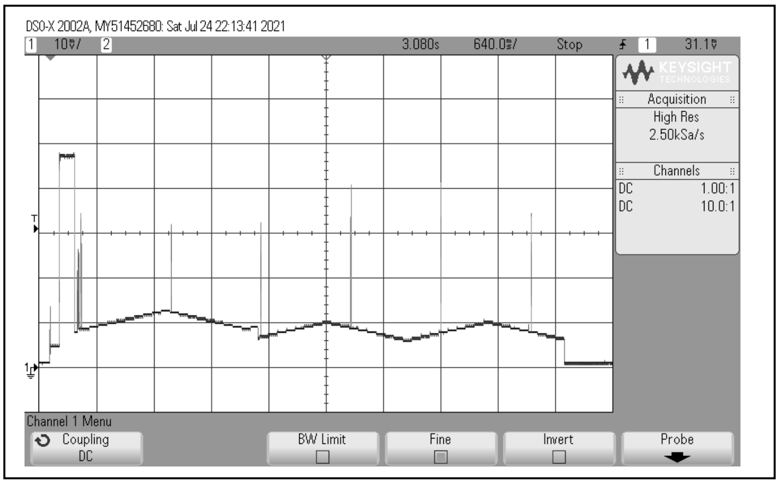

Figure S5. Start-up sequence measured when the Flower Care™ is turned on.

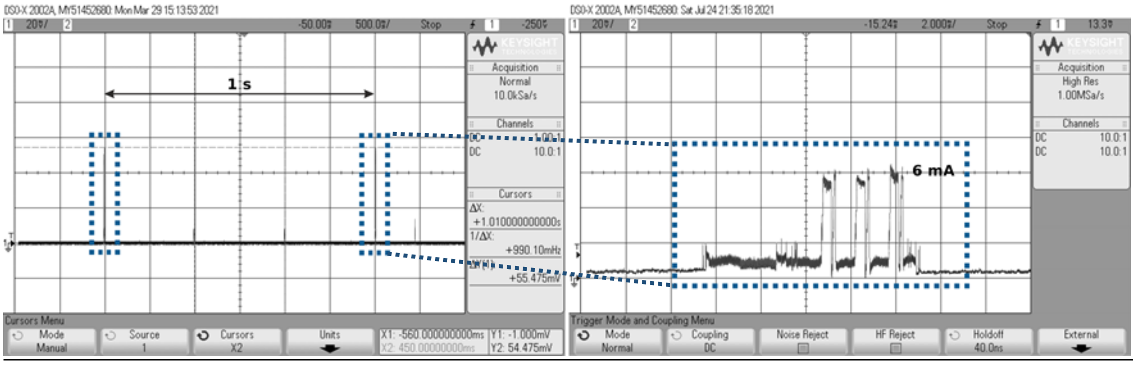

Figure S6. Advertising mode: this mode runs continuously in background, it helps the cell phone to detect the sensor during scanning for connection (the sensing is in sleep mode between these peaks). Right: advertising interval between each advertising event is found to be 1 s. Left: magnified view of one peak.

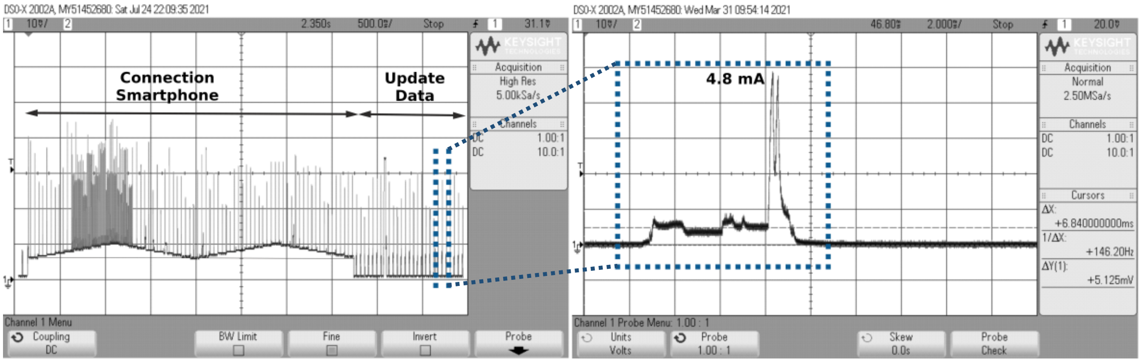

Figure S7. Connection mode. Left: connection peaks/events occur every 45 ms. This mode stays active as long as the gardener tries to update data on the dedicated app. Right: enlarged view of one peak.

Supplementary References

1 S. Aslam, F. Farooq and S. Sarwar, *Proc. 6th Int. Conf. Front. Inf. Technol. FIT '09*, , DOI:10.1145/1838002.1838017.

2 B.H.E. Vanthoor, P.H.B. de Visser, C. Stanghellini, E.J. van Henten, *A methodology for model-based greenhouse design: Part 2, description and validation of a tomato yield model*, DOI:10.1016/j.biosystemseng.2011.08.005.
